# Radiotherapy and survival in elderly grade 4 glioma patients: The prognostic value of onco-functional outcome

**DOI:** 10.1016/j.ctro.2025.101085

**Published:** 2025-11-20

**Authors:** Helen X. Hou, Sophia M. Leiss, Daniel Schmottermeyer, Christian Diehl, Benedikt Wiestler, Jan Peeken, Kai Borm, Chiara Negwer, Arthur Wagner, Igor Yakushev, Claire Delbridge, Meike Mitsdoerffer, Friederike Schmidt-Graf, Bernhardt Meyer, Stephanie Combs, Denise Bernhardt

**Affiliations:** a Department of Radiation Oncology, Klinikum rechts der Isar der TU München, Ismaninger Straße 22, 81675 Munich, Germany; b Department of Neuroradiology, Klinikum rechts der Isar der TU München, Munich, Germany; c TranslaTUM, TU Munich, 81675 München, Germany; d Department of Neurosurgery, Klinikum rechts der Isar der TU München, Munich, Germany; e Department of Nuclear Medicine, Klinikum rechts der Isar der TU München, Munich, Germany; f Department of Pathology and Neuropathology, TUM School of Medicine, TU Munich, 81675 München, Germany; g Department of Neurology, Klinikum rechts der Isar der TU München, Munich, Germany; h Institute of Radiation Medicine (IRM), Department of Radiation Sciences (DRS), Ingolstädter Landstraße 1, Neuherberg, Germany; i Deutsches Konsortium für Translationale Krebsforschung (DKTK), Partner Site Munich, Germany

**Keywords:** Glioma, Prognostic Score, Onco-Functional Outcome, Radiotherapy

## Abstract

**Purpose:**

**/Objectives:** Older adults with WHO grade 4 glioma face distinct therapeutic challenges due to age-related comorbidities, treatment intolerance and tumor aggressiveness. This retrospective study evaluates the prognostic value of the Onco-Functional Outcome (OFO) classification, which integrates extent of resection and postoperative functional status, in the context of radiotherapy (RT) for patients with grade 4 glioma.

**Materials:**

**/Methods:** 139 patients aged ≥ 60 years treated between 2001 and 2021 were included. Patients were grouped into four OFO categories based on Karnofsky Performance Status, National Institutes of Health Stroke Scale, and resection extent. Survival outcomes were assessed using Kaplan-Meier and compared by log-rank test. Univariate and multivariable analyses were performed using Cox proportional hazards model.

**Results:**

Median OS decreased across OFO groups (OFO1-4: 22.6, 20.5, 12.6, and 11.2 months respectively, log-rank, p = 0.0026). PFS followed a similar pattern (12.2, 9.3, 6.7, and 5.7 months respectively; p = 0.0030). Among 119 patients with known MGMT status, methylation (n = 49) was associated with significantly prolonged OS (36.3 vs. 12.6 months, p < 0.00001) and PFS (14.1 vs 5.9 months, p < 0.00001). Survival advantage of NF–RT showed a a strong trend in both univariate (OS HR = 0.47, 95 % CI 0.28–0.82; p = 0.007) and multivariate models (OS HR = 0.57, 95 % CI 0.33–1.00, 0.051). In multivariable analysis, the OFO classification retained independent prognostic value, with OFO Group 4 associated with a significantly increased risk of both death (OS: HR = 2.91, p = 0.003) and disease progression (PFS: HR = 3.08, p = 0.0005) compared to OFO Group 1.

**Conclusions:**

OFO classification effectively stratifies survival in elderly grade 4 glioma patients. Its integration with MGMT methylation status and RT regimen refines risk assessment, supporting personalized treatment strategies in this vulnerable patient group.

## Highlights

• OFO classification meaningfully stratifies OS and PFS in elderly grade 4 glioma patients.
• MGMT methylation prognostic value shows significance across OFO subgroups.
• Normofractionated-RT improves OS in vulnerable OFO 4 patients compared to hypofractionated-RT.

## 1 Introduction

WHO grade 4 gliomas represent the most aggressive and fatal class of primary brain tumors in adults, characterized by rapid progression, treatment resistance, and poor survival outcomes [1]. These tumors include IDH-wildtype glioblastoma (GBM) and IDH-mutant astrocytomas with grade 4 histologic features, as redefined in the 2021 WHO CNS tumor classification [2]. Older adults with grade 4 glioma represent a clinically distinct population, often facing age related physiological decline, comorbidities and reduced tolerance to standard therapies [3,4]. Yet there is no consensus across literature on what age defines “elderly” in this contect. While various studies use cutoffs of 60 or 65 years, others including the European Association of Neuro-Oncology (EANO) guidelines, recommend higher thresholds such as 70 or 75 years [[5], [6], [7], [8]].

The current standard of care consists of maximal safe resection followed by radiotherapy (RT) and temozolomide chemotherapy (Stupp-protocol), yet outcome remains poor, with median OS ranging from 12-18 months and a 5 year survival rate of only 4.1 % [9,10]. The role of gross total resection (GTR) in improving survival in glioma patients has been extensively studied [7,11], its benefit in older patients however, is less clear. Similarly, RT regimen for this population remains debated. Standard normofractionated (NF)-RT- 60 Gy in 30 fractions, is often associated with increased treatment burden and toxicity in elderly and frail patients. Consequently, hypofractionated (HF)-RT regimens (e.g., 40 Gy in 15 fractions) have been explored as alternatives to improve treatment tolerability while maintaining efficacy [12]. This diversity in RT approaches highlights the importance of stratified analysis to better understand age-related effects on treatment efficacy and toxicity.

To address these questions, we apply the Onco-Functional Outcome (OFO) classification, recently introduced by Gerritsen et al., to a retrospective cohort of older adults with WHO grade 4 glioma [13]. The OFO framework categorizes patients based on the extent of tumor resection and postoperative functional status grouping them into four distinct classes, OFO 1–4 [13]. In this study, we assess the prognostic value of OFO and its interaction with MGMT promoter methylation status and RT regimen. Additionally we perform age subgroup analyses to further refine risk stratification and guide personalized treatment strategies for this high-risk population.

## 2 Materials and methods

### 2.1 Patient cohort

We retrospectively analyzed 139 patients with histologically confirmed WHO grade 4 glioma treated with adjuvant RT between 2001 and 2021 at the Department of Radiation Oncology, TUM University Hospital. Eligibility criteria included histopathological diagnosis and available pre- and postoperative functional assessments. Electronic medical records were reviewed for baseline demographic data, disease characteristics, RT treatment details, and acute toxicities according to the common terminology criteria for adverse events, CTCAEv5.0. Functional status was assessed using the Karnofsky Performance Status (KPS) and the National Institutes of Health Stroke Scale (NIHSS), with postoperative evaluations performed 6 weeks after resection and prior to RT initiation. Demographic cohort data were summarized using descriptive statistics (Table 1).

**Table 1:** Patient and RT treatment characteristics.

**Characteristic**	**OFO 1**	**OFO 2**	**OFO3**	**OFO 4**
**Total**	N = 31	N = 29	N = 34	N = 45
**Gender**				
Male	18 (58.1 %)	12 (41.4 %)	21 (61.8 %)	22 (48.9 %)
Female	13 (41.9 %)	17 (58.6 %)	13 (38.2 %)	23 (4.4 %)
**Age at diagnosis**				
Mean (SD)	68.2 (5.8)	69.9 (5.3)	71.5 (7.8)	70.9 (7.2)
Median [Min, Max]	66.4 [61.2–82.3]	69.5 [61.1–82.1]	71.3 [61.6–85.5]	68.0 [61.2–85.5]
**Preoperative KPS**				
Mean (SD)	77.1 (14.4)	68.3 (14.7)	73.2 (16.1)	77.1 (13.6)
Median [Min, Max]	80 [40–100]	70 [40–100]	70 [50–100]	80 [50–100]
**Preoperative NIHSS**				
Mean (SD)	1.4 (1.5)	2.1 (2.5)	1.5 (1.6)	1.7 (1.3)
Median [Min, Max]	1 [0–6]	1.0 [0–9]	1 [0–6]	2 [0–4]
**Resection status**				
GTR	31 (100 %)	0 (0 %)	34 (100 %)	0 (0 %)
STR	0 (0 %)	29 (100 %)	0 (0 %)	45(100 %)
**MGMT status (total 119)**				
Methylated	12/49 (24.5 %)	13/49 (25.5 %)	7/49 (14.3 %)	17/49 (34.7 %)
Unmethylated	16/70 (22.9 %)	11/70 (15.7 %)	22/70 (31.4 %)	21/70 (30.0 %)
**IDH status**				
Wildtype	26/27 (96.3 %)	23/26 (88.5 %)	28/29 (96.6 %)	37/41 (90.2 %)
Mutant	1/27 (3.7 %)	3/26 (11.5 %)	1/29 (3.4 %)	4/41 (9.8 %)
**Time Resection to RT (months)**				
Mean (SD)	1.2 (0.6)	0.82 (2.08)	1.33 (1.72)	0.9 (2.6)
Median	1.1 [0.5, 3.2]	0.9 [-9.2, 3.65]	0.89 [0.5, 10.5]	0.9 [-11.33, 12.78]
**Fractionation**				
Normofractionated RT	26 (83.9 %)	19 (65.6 %)	16 (47.1 %)	20 (44.4 %)
Hypofractionated RT	5 (16.1 %)	10 (34.5 %)	18 (52.9 %)	25 (55.6 %)
**Fraction**				
Mean (SD)	26.87 (7.15)	26.07 (5.8)	22.5 (7.1)	21.71 (7.47)
Median	30 [1, 33]	30 [14, 30]	23 [14, 30]	23 [10, 33]
**Dose (Gy)**				
Mean (SD)	55.5 (8.5)	53.6 (8.1)	49.7 (9.3)	47.8 (9.7)
Median (Min, Max)	60.0 [30.0–66.0]	60.0 [40.1–60.0]	46.4 [37.4, 60.0]	43.9 [20.0–60.0]

### 2.2 OFO classification

Patients were classified into OFO groups according to Gerritsen et al[13]. The OFO Score integrates the extent of resection and functional loss, defined as KPS deterioration of ≥ 10 points in or ≥ 1 point NIHSS deterioration at 6 weeks post-op. Extent of resection was categorized according to the Response Assessment in Neuro-Oncology (RANO) classification, with gross-total resection (GTR) defined as maximum contrast-enhancing resection (RANO class 2) corresponding to a residual tumor volume of ≤ 1 mL [14]. OFO categories included: OFO 1 (GTR with no functional loss), OFO 2 (subtotal resection (STR) with no functional loss), OFO 3 (GTR with functional loss), and OFO 4 (STR with functional loss)[13].

### 2.3 Statistical methods

The primary study endpoint was overall survival (OS), secondary endpoints included progression free survival (PFS) and acute toxicity. Survival outcomes were assessed using Kaplan-Meier estimates, and the relationship between OFO groups and survival was analyzed using the log-rank test and Cox regression. OS and PFS were defined as the time from the date of first resection to the respective event. Patients without an event were censored at the date of last follow-up. Univariable and multivariable Cox proportional-hazards (PH) regression was performed. Variables that influence survival in the univariable testing were then included in the multivariable Cox PH model. Hazard ratios (HR) with two-sided 95 % confidence intervals (CI) and p-values were calculated. P values < 0.05 were considered statistically significant. Statistical analyses were performed using Python (v3.13.1). Data manipulation and processing were performed using pandas (v2.2.3), lifelines (v0.30.0) were employed for survival analysis and matplotlib was used for data visualization.

## 3 Results

139 patients with a median age of 69.2 years at the time of first resection were available for analysis. We examined the OS across the four OFO groups derived from combined KPS and NIHSS scores.

### 3.1 Overall cohort

**Fig. 1:**
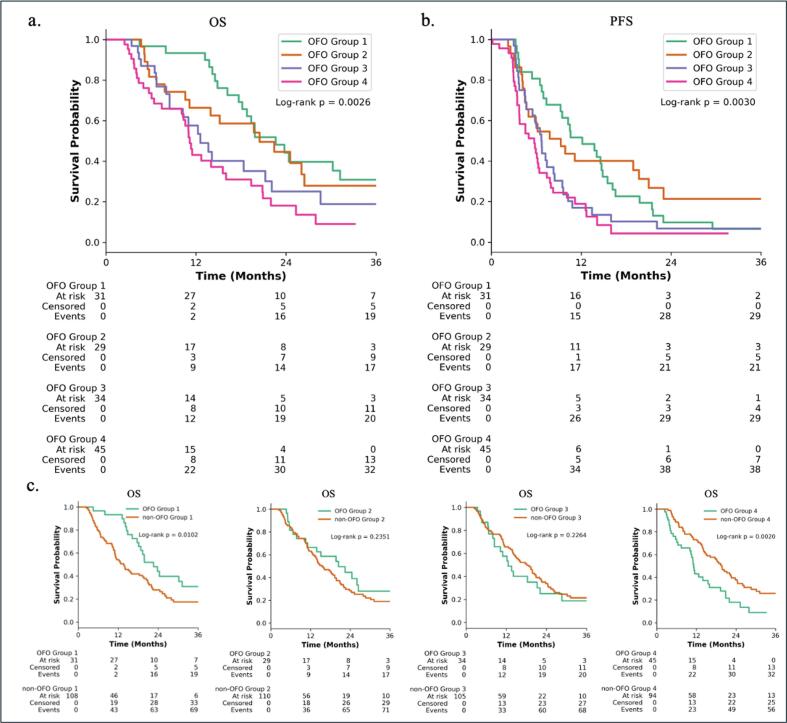
Kaplan-Meier survival plot. (a) OS by OFO group 1–4, demonstrating a stepwise decline in OS. (b) PFS by OFO group, similarly showing shorter PFS in higher OFO categories. (c) OS comparison between each OFO group and their non-OFO counterparts. P-values, log-rank test.

In the overall cohort, Kaplan-Meier analysis revealed distinct differences among the four OFO groups. Median OS decreased with increasing OFO group: OFO 1 had a median OS of 22.6 months, followed by OFO 2 (20.5 months), OFO 3 (12.6 months), and OFO group 4 (11.2 months), log-rank, p = 0.0026 (Fig. 1a). Similarly, median PFS declined across the groups, from 12.2 months in OFO group 1 to 9.3, 6.7, and 5.7 months in OFO groups 2, 3, and 4, respectively (log-rank p = 0.0030) (Fig. 1b).

To further explore the prognostic relevance of each OFO subgroup, we conducted pairwise Kaplan-Meier survival analyses comparing patients within each OFO group to all other OFO groups (OFO group *n* vs. non-OFO group *n*) (Fig. 1c): OFO 1 demonstrated significantly improved survival compared to non-OFO group 1 (median OS 22.6 vs 13.6 months, log-rank p = 0.010). While OFO 4, which reflects the worst scenario for the patient (both functional loss and incomplete resections), was associated with significantly poorer outcomes. Median OS in OFO 4 was 11.2 months, compared to 19.7 months in non-OFO 4 (log-rank, p = 0.002).

### 3.2 Methylation status

**Fig. 2:**
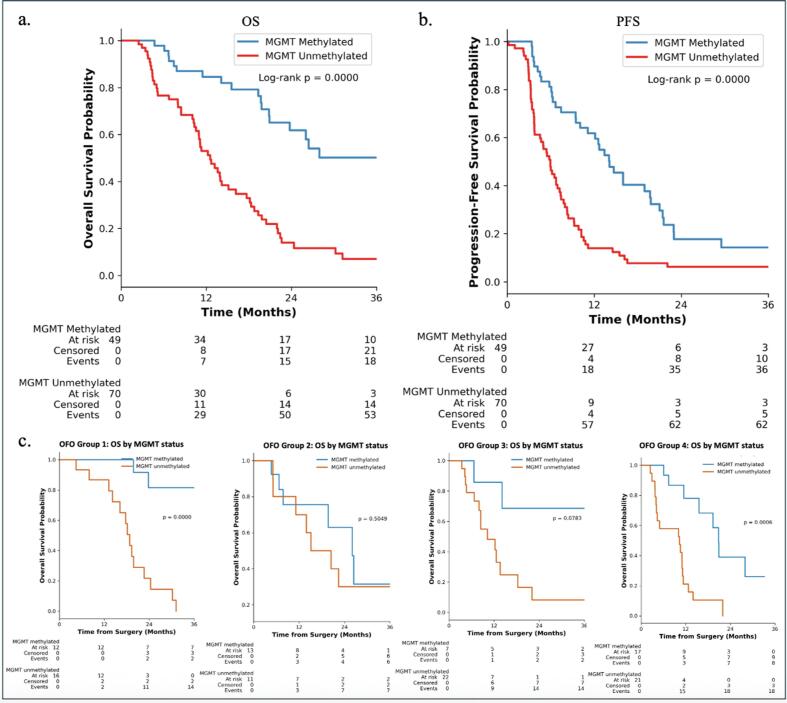
Kaplan-Meier survival plots. (a) OS stratified by MGMT status. (b) PFS stratified by MGMT status (c) Subgroup analysis of OS by OFO group and MGMT OS comparison between each OFO group and their non-OFO counterparts in P-values, log-rank test.

In a subset of 119 patients with known MGMT promoter methylation status, n = 49 (41.22 %) were classified as methylated and n = 70 (58.8 %) as unmethylated. Patients with MGMT methylation had markedly prolonged OS and PFS compared to MGMT unmethylated patients (median OS 36.3 vs 12.6, median PFS 14.1 vs 5.9 months; log-rank, p < 0.00001) (Fig. 2a + b). To examine whether MGMT promoter methylation conferred differential survival benefit across OFO groups, we carried out subgroup specific Kaplan-Meier analyses. Survival advantage associated with MGMT methylation was most pronounced in OFO 1 and OFO 4, whereas no significant differences were seen in OFO 2 and OFO 3 (see Fig. 2c for details).

### 3.3 Radiation therapy regimen

**Fig. 3:**
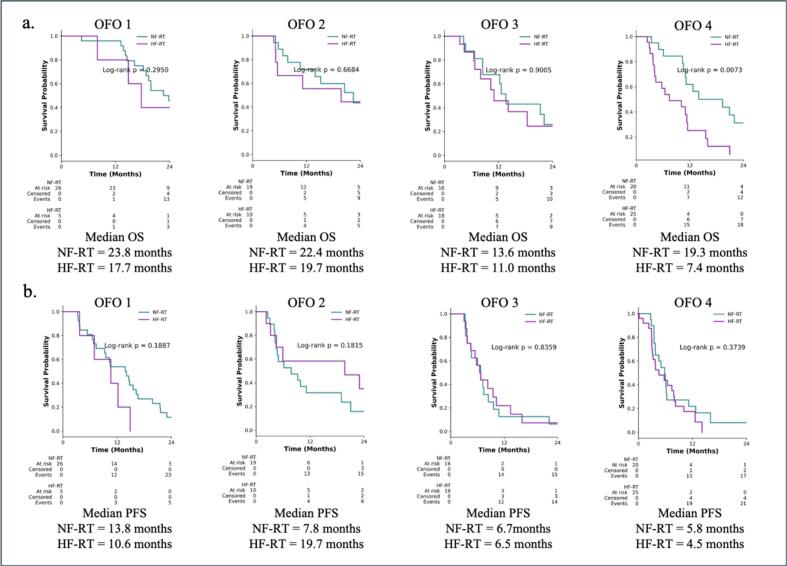
Kaplan-Meier survival plot. (a) OS by OFO group 1–4, demonstrating a stepwise decline in OS. (b) PFS by OFO group, similarly showing shorter PFS in higher OFO categories. P-values, log-rank test.

RT Treatment characteristics are summarized in Table 1. The median time from resection to RT initiation was consistent across groups, ranging from 0.9-1.1 months. The proportion of patients receiving HF-RT increased across OFO groups 1–4 (OFO 1: n = 5, OFO2 n = 10, OFO3 n = 18, OFO 4n = 25). Mean total RT doses also declined with increasing OFO group, from 55.5 Gy in OFO1, 53.6 Gy in OFO2, 49.7 Gy in OFO3, to 47.8 Gy in OFO4. Across all OFO groups, patients receiving NF-RT consistently demonstrated longer median OS compared to those treated with HF-RT. In OFO Group 1, the median OS was 23.8 months with NF-RT versus 17.7 months with HF-RT (log-rank p = 0.2950). In OFO Group 2, median OS was 22.4 months versus 19.7 months (p = 0.6684); in OFO Group 3, 13.6 months versus 11.0 months (p = 0.9005). Notably, a statistically significant survival advantage for NF-RT was observed in OFO Group 4, where median OS reached 19.3 months compared to 7.4 months with HF-RT (log-rank p = 0.0073). Across all four OFO groups (OFO1-4), patients receiving NF-RT tended to experience a numerically longer PFS than those treated with HF-RT, although the difference did not reach statistical significance in any group (Fig. 3).

Of the 139 patients included, 27 (19.4 %) developed motor deficits, 13 (13.7 %) experienced sensory disturbances, and 14 (10.1 %) had new-onset epilepsy following treatment. Hematologic toxicities included leukopenia in 9 patients (6.4 %) and thrombocytopenia in 15 patients (10.8 %). Other non-specified treatment-related toxicities were reported in 107 cases (77.0 %) within the first six months following radiotherapy initiation.

### 3.4 Age

**Fig. 4:**
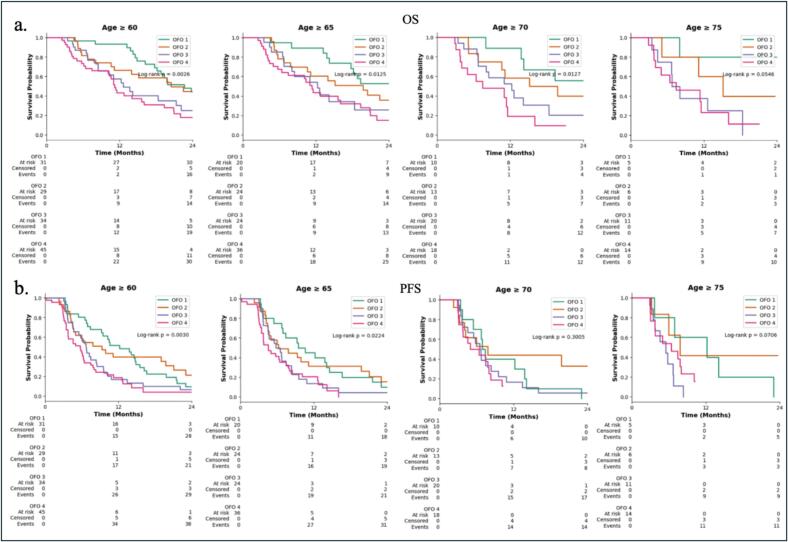
Kaplan-Meier survival plot. (a) OS by OFO group 1–4 stratified by age groups. (b) PFS by OFO group stratified by age groups. P-values, log-rank test.

Recognizing the variability in age definitions for elderly patients across glioma studies, we conducted subgroup analyses to assess the applicability of OFO classification across different age thresholds ((≥60, ≥65, ≥70, ≥75 years) (Fig. 4). For OS significant differences between OFO groups were observed in patients aged ≥ 60 years (p = 0.0026), ≥65 years (p = 0.0125), and ≥ 70 years (p = 0.0127). In patients aged ≥ 75 years, the difference trended toward statistical significance (p = 0.0546). For PFS, the OFO classification significantly discriminated outcomes in patients aged ≥ 60 years (p = 0.0030) and ≥ 65 years (p = 0.0224). However, OFO assignment were no longer statistically significant in patients aged ≥ 70 years (p = 0.3005) and ≥ 75 years (p = 0.0706).

### 3.4 Univariable and multivariable analysis of OS

In univariate Cox PH analyses (Table 2), MGMT promoter methylation had a strong impact on OS (HR = 0.24, 95 % CI 0.13–0.43, p < 0.001), as was the receipt of NF-RT (HR = 0.47 95 % CI 0.28–0.82, p = 0.007). Among the OFO groups – using OFO 1 as the reference group, only OFO 4 (subtotal resection + functional decline) was linked to poorer OS (HR = 1.80, 95 % CI 1.02–3.17, p = 0.004). Postoperative deterioration in KPS had no impact on OS, while NIHSS deterioration showed a non–significant trend toward worse survival (HR 1.70, 95 % CI 0.98–2.94, p = 0.059). Neither age at diagnosis nor STR reached significance in the univariable setting (all p > 0.05).

**Table 2:** Uni- and Multivariate Model of Overall Survival.

**Univariate Model**			
**Variable**	**HR**	**95 % CI**	**p-value**
Age at First Diagnosis	1.03	0.99 – 1.07	0.177
MGMT methylated	0.24	0.13 – 0.43	< 0.001
NF-RT	0.47	0.28 – 0.82	0.007
STR	1.3	0.78 – 2.17	0.317
KPS deterioration of ≥ 10 points	1.01	0.59 – 1.71	0.976
NIHSS deterioration of ≥ 1 point(s)	1.7	0.98 – 2.94	0.059
OFO 2	1.09	0.56 – 2.10	0.801
OFO 3	1.46	0.77 – 2.79	0.25
OFO 4	1.8	1.02 – 3.17	0.004
**Multivariate Model**			
**Variable**	**HR**	**95 % CI**	**p-value**
MGMT methylated	0.2	0.11 – 0.38	< 0.001
NF-RT	0.57	0.33 – 1.00	0.051
OFO 2	1.48	0.67 – 3.25	0.329
OFO 3	2.5	1.12 – 5.54	0.025
OFO 4	2.91	1.43 – 5.91	0.003

In the multivariate analysis adjusting for MGMT status, RT regimen and OFO classification (with OFO 1 again as the reference), MGMT promoter methylation remained the strongest independent predictor of prolonged OS (HR = 0.20, 95 % CI 0.11–0.38, p < 0.001). NF-RT showed a strong trend towards improved OS (HR = 0.57, 95 % CI 0.33–1.00, p = 0.051), but did not reach statistical significance. Compared to OFO 1, both OFO 3 (GTR with postoperative functional decline) and OFO 4 (STR and postoperative functional decline) demonstrated significantly increased risk of death (OFO 3: HR 2.50, 95 % CI 1.12–5.54, p = 0.025; OFO 4: HR 2.91, 95 % CI 1.43–5.91, p = 0.003), whereas OFO 2 did not reach statistical significance (HR 1.48, 95 % CI 0.67–3.25, p = 0.329).

### 3.5 IDH-wildtype

IDH-wildtype GBM represent a distinct molecular entity defined by the absence of mutations in the IDH1 or IDH2 genes, and are known to be associated with more aggressive biological behavior compared to their IDH-mutant counterparts [15,16]. To assess whether the presence of IDH-mutant cases influenced survival outcomes within the OFO subgroups, all IDH-mutant patients (n = 9) were excluded and analyses were repeated in the IDH-wildtype cohort. Survival differences among the four OFO groups remained significant. Median OS declined with increasing OFO group: median OS was 22.6 months for OFO 1, 19.7 months for OFO 2, 12.6 months for OFO 3, and 11.0 months for OFO 4 (log-rank p = 0.0004) (Supplement Fig. S1). Median PFS similarly decreased across groups, from 10.6 months in OFO group 1 to 7.8, 6.7, and 5.0 months in OFO groups 2, 3, and 4 respectively (log-rank 0.0007). Additional subgroup analyses of MGMT promoter methylation status and RT regimen (NF-RT vs HF-RT) did not reveal significant differences after exclusion of IDH-mutant cases (Supplement Fig. S2, S3).

## 4 Discussion

In this retrospective cohort study, we validated the OFO classification. A novel framework initially proposed by Gerritsen et al. in 2024, which integrates functional performance (via KPS and NIHSS) and surgical extent into a single composite score. Proposed as a stratification factor for therapeutic neuro-oncological trials, the OFO system can offer a pragmatic tool to guide clinical decision-making. We extend its application by stratifying survival outcomes across RT regimens in GBM patients aged ≥ 60 years, demonstrating how OFO classification can inform not only prognosis but also therapeutic planning.

Our findings confirm that OFO classification significantly stratifies patient outcomes. Patients in OFO 1 achieving GTR without postoperative deficits experienced the longest median OS (22.6 months) and PFS (12.2 months), while those in OFO 4, characterized by both incomplete resection and postoperative functional decline, had the poorest outcomes (median OS 11.2 months and PFS 5.7 months). Importantly, pairwise comparisons of each OFO group against their non-OFO counterparts reinforced these gradients, with OFO 1 showing a statistically significant survival advantage (p = 0.010) and OFO 4 markedly poorer outcomes (p = 0.002). These findings align with previous published data indicating that both postoperative neurological status and resection extent are both key prognostic factors in GBM [17], [18]. Notably, OFO 2 patients, who had STRs but no deficits, did better than OFO 3 patients that had GTRs but postoperative functional decline (median OS 20.5 vs. 15.6 months, p = 0.235), suggesting that in our cohort, as in Gerritsen et al., preservation of function may outweigh the survival benefit conferred by extent of resection alone. In multivariable analysis, OFO grouping remained an independent and robust predictor of OS: OFO 3 was associated with a 2.50–fold increased risk of death (HR = 2.50; 95 % CI 1.12–5.54; p = 0.025) and OFO 4 with a 2.91–fold increased risk (HR = 2.91; 95 % CI 1.43–5.91; p = 0.003), each compared to OFO 1. OFO 2 did not differ significantly from OFO 1 (HR = 1.48, p = 0.33). Notably the emergence of OFO 3 as significant only in multivariate analysis as compared to univariate analysis suggests that its adverse impact become clearer once molecular and RT regimen factors are accounted for. These results position the OFO classification as a meaningful complement to existing molecular markers such as MGMT methylation status.

MGMT promoter methylation status was confirmed as a strong and independent prognostic molecular marker in our cohort, significantly associated with prolonged OS (HR = 0.20, p < 0.001). This is in line with previous evidence demonstrating the predictive value of MGMT methylation for treatment response to alkylating agents such as temozolomide in GBM patients [7,19,20]. Importantly, our subgroup analysis reveals that the survival advantage conferred by MGMT methylation is modulated by the patient's OFO group: while OFO 1 and 4 showed a significant survival benefit in methylated versus unmethylated patients (median OS 71.6 vs. 18.8 months, p < 0.00001, and median OS 20.9 vs 10.3 months, p = 0.0006, respectively). In contrast, this effect decreased in OFO group 2 and 3 and did not reach statistical significance, although a trend was observed in OFO group 3 (MGMT methylated: 40.9 months vs. MGMT unmethylated: 10.2 months, p = 0.0783). One possible explanation is that in patients with the most favorable (OFO 1) or least favorable (OFO 4) status, the impact of tumor biology such as MGMT methylation on outcome is more clearly expressed. In OFO 1, longer survival times allow the treatment benefit from temozolomide to fully manifest, while in OFO 4, even modest therapeutic benefit translate into significant differences due to otherwise poor prognosis. In intermediate groups OFO 2 and 3, other competing factors such as comorbidities, surgical extent and impairments may have increased influence on the predictive power of MGMT methylation. Additionally, smaller subgroup sizes may have limited statistical power to detect differences in these groups.

[15,16]. While we recognize that these patients – comprising 6.5 % of our cohort and more prevalent in certain OFO subgroups – have superior survival outcomes, the limited number of IDH mutant cases in our study precluded meaningful subgroup or sensitivity analyses. To examine whether the presence of IDH-mutant astrocytomas influenced our main findings, we performed separate analyses of the IDH-wildtype patients. The results demonstrated that OFO group differences in outcomes remained significant and robust. There were also no significant changes observed for MGMT methylation or RT regimen subgroup analyses. However, we acknowledge the small number of IDH-mutant cases in our cohort overall, which limits the power to detect effects attributable to this subgroup. Future studies with larger cohorts should control or adjust for IDH mutational status to further clarify these effects.

Recognizing the heterogeneity in how elderly glioma patients are defined across studies, we evaluated the discriminatory capacity of the OFO classification across multiple age threshold (≥60, ≥65, ≥70, and ≥ 75 years). Our results demonstrate that the OFO system retains prognostic value for both OS and PFS in patients aged ≥ 60 and ≥ 65, but its discriminatory power diminished in older subgroups. Specifically, significant differences between OFO groups were observed for OS in patients ≥ 60, ≥65, and ≥ 70 years, with a strong trend toward significance in those ≥ 75 years. For PFS, the significance was lost in patients ≥ 70 and ≥ 75 years prognostic value is strongest when “elderly” is defined more inclusively. These observations align with prior reports by Gerritsen et al. who noted that in those ≥ 70 years only OFO 1 patients derived a significant survival benefit, whereas other OFO strata converged. Several factors may explain this attenuation in prognostic separation in the oldest cohorts. First, the number of patients ≥ 70 and ≥ 75 was markedly lower, limiting statistical power to detect survival differences. Second, advancing age itself is a dominant prognostic factor in grade 4 glioma patients, outweighing the contribution of surgical and functional parameters in the OFO classification. Older patients are also more likely to experience higher treatment-related toxicitiy, reduced tolerance to multimodality treatment, and competing risks from comorbidities, all of which may blur the survival distinctions captured by OFO categories. These findings combined indicate that while OFO remains a valuable tool in glioma patients, its predictive capacity diminishes with advancing age. It underscores the need to integrate geriatric specific factors for individualized risk assessment in patients ≥ 75 years.

Our findings demonstrate that the benefit of RT in grade 4 glioma patients is modulated by the patient's postoperative functional and surgical status as captured by the OFO classification. Across all OFO groups, patients who received NF-RT consistently exhibited numerically longer OS than those treated with HF-RT, although these differences reached statistical significance only in OFO 4 (OS: 19.3 vs. 7.4 months, p = 0.0073). Patients in OFO 4 represent a particularly vulnerable subgroup with both postoperative functional deterioration and incomplete resection. In this group, the observed survival benefit associated with NF-RT suggests that more intensive RT regimens may help offset poor surgical and functional baseline conditions. However, this potential advantage in OFO group 4 must be weight very carefully, as patients with poor baseline status require precise evaluation to ensure that the risks and treatment-related toxicities of more intensive RT do not outweigh its benefits. Notably, the survival advantage of NF–RT persisted as a strong trend in both univariate (OS HR = 0.47, 95 % CI 0.28–0.82; p = 0.007) and multivariate models (OS HR = 0.57, 95 % CI 0.33–1.00, 0.051). The fact that no statistically significant differences in PFS were observed across OFO groups may reflect limitations in power or follow-up time but also underscores the complexity of functional status as a modifier of RT efficacy.

Importantly, interpretation of outcomes related to RT fractionation regimens is limited by the small sample size in each subgroup and the potential selection bias inherent to retrospective studies. Nonetheless, these results provide valuable insights and reflect real-world data from routine clinical settings. To our knowledge, this study is among the first to apply the OFO framework into comparative RT outcome analysis, highlighting its potential value for refining clinical decision-making on RT treatment intensification or de-escalation.

## 5 Conclusion

This study independently validates the OFO classification as a clinically relevant prognostic tool for patients with grade 4 glioma, highlighting its utility in radiation oncology by combining postoperative functional status and extent of resection into a single framework that supports individualized treatment decisions. OFO grouping demonstrated robust and independent predictive value for both OS and PFS, outperforming traditional functional scores in multivariable analysis. Importantly, our data highlight that the impact of postoperative functional deficits may outweigh the benefit of complete resection alone, highlighting the principle of maximal safe resection rather than maximal resection. Furthermore, we demonstrate for the first time that OFO classification meaningfully stratifies outcomes in relation to RT fractionation strategies, with normofractionated RT conferring the most benefit in patients with the poorest postoperative status (OFO 4) and outcome. Together, these findings position OFO as a useful adjunct to molecular classifiers such as MGMT methylation, with potential to guide individualized therapeutic decision-making and improve risk stratification in both clinical practice and trial design.

## Availability of data and materials

The datasets analyzed during the current study are not publicly available due to patient privacy and institutional restrictions but are available from the corresponding author on reasonable request.

## Authors' contributions

DB conceptualized the topic of this work. DB and SC obtained ethical approval. HH and SL wrote the manuscript. DS, BW, CD, JP, KB, CN, AW, IY, CD, MM, FS, and BM provided patient data. Statistical analysis was conducted by HH. SL and BW contributed to interpretation of the data. DB is the primary investigator. All authors read, edited, and approved the final manuscript.

## Ethics approval

This study was approved by the ethics committee of the Technical University of Munich, Germany on July 13, 2023 (2021-676_1-S). The requirement of informed consent was waived by the ethical committee due to the retrospective nature of this study. All examinations and evaluations were performed following institutional guidelines and the Declaration of Helsinki of 1975 in its most recent and updated version.

## Funding

This research did not receive any specific grant from funding agencies in the public, commerical, or not-for-profit sectors.

## Declaration of competing interest

The authors declare that they have no known competing financial interests or personal relationships that could have appeared to influence the work reported in this paper.
