# Supplementary material for: Radiotherapy and survival in elderly grade 4 glioma patients: The prognostic value of onco-functional outcome
Source: Clin Transl Radiat Oncol. 2025 Nov 20;56:101085. doi: 10.1016/j.ctro.2025.101085 (PMC12681765; doi:10.1016/j.ctro.2025.101085)
Supplement: Supplementary Data 1 [file mmc1.docx]

**Supplementary Material**

Figure S1

**
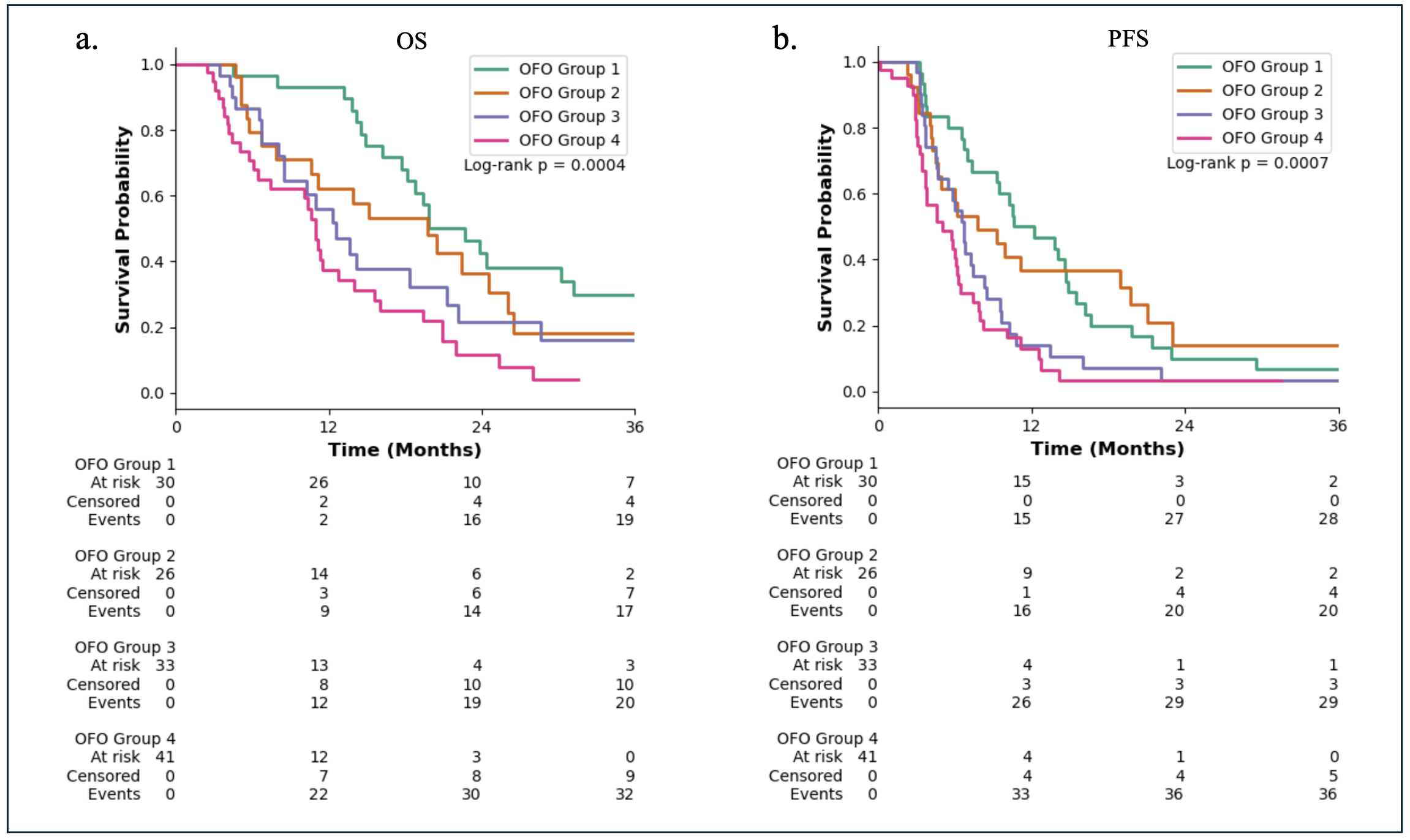
**

**Figure S1.** Kaplan-Meier survival plot of IDH-wildtype GBM. (a) OS by OFO group 1-4, demonstrating a stepwise decline in OS. (b) PFS by OFO group, similarly showing shorter PFS in higher OFO categories.

P-values, log-rank test.


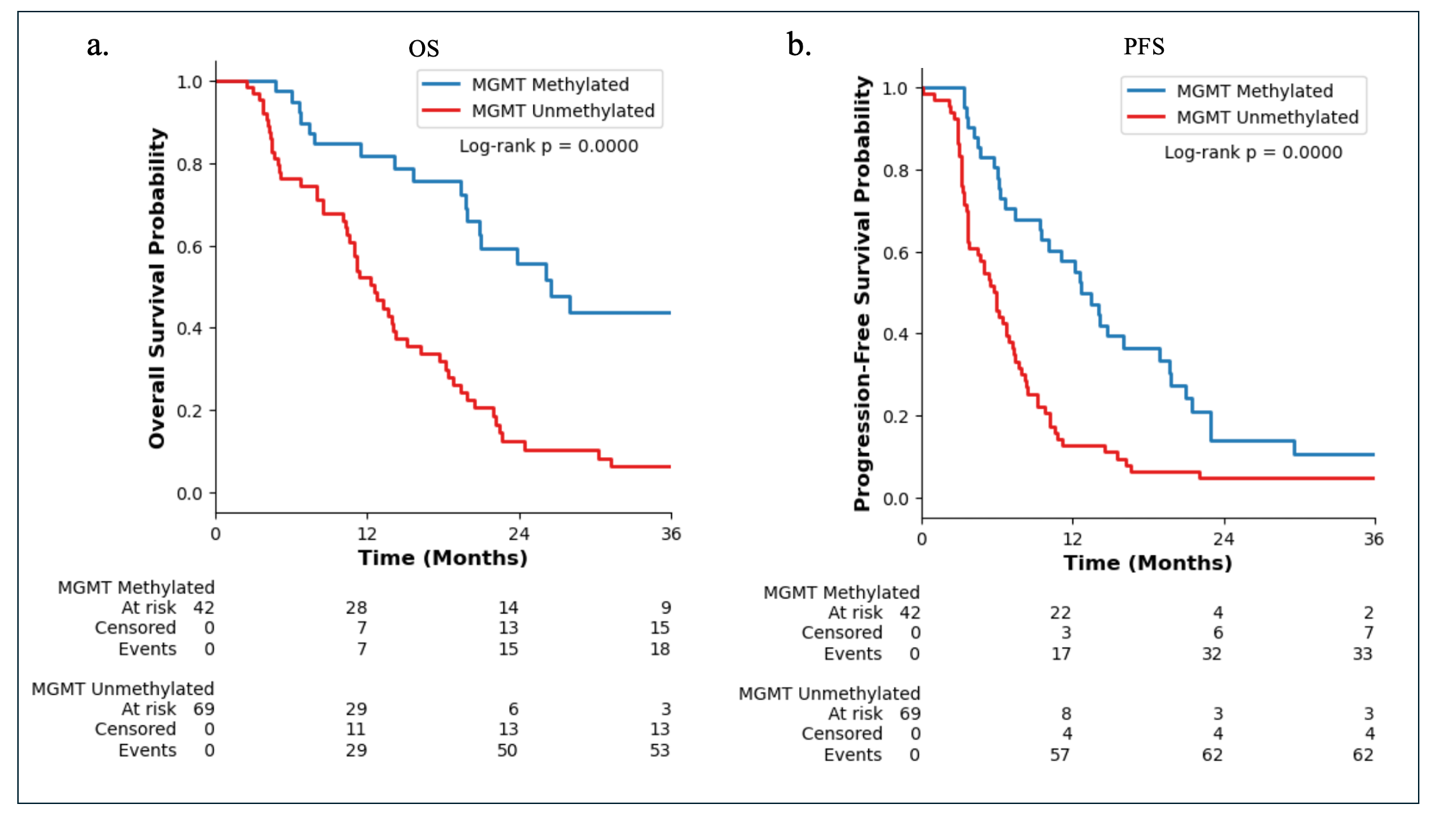
Figure S2

**Figure S2.** Kaplan-Meier survival plots of IDH-wildtype GBM. (a) OS stratified by MGMT status. (b) PFS stratified by MGMT status. P-values, log-rank test.

Figure S3


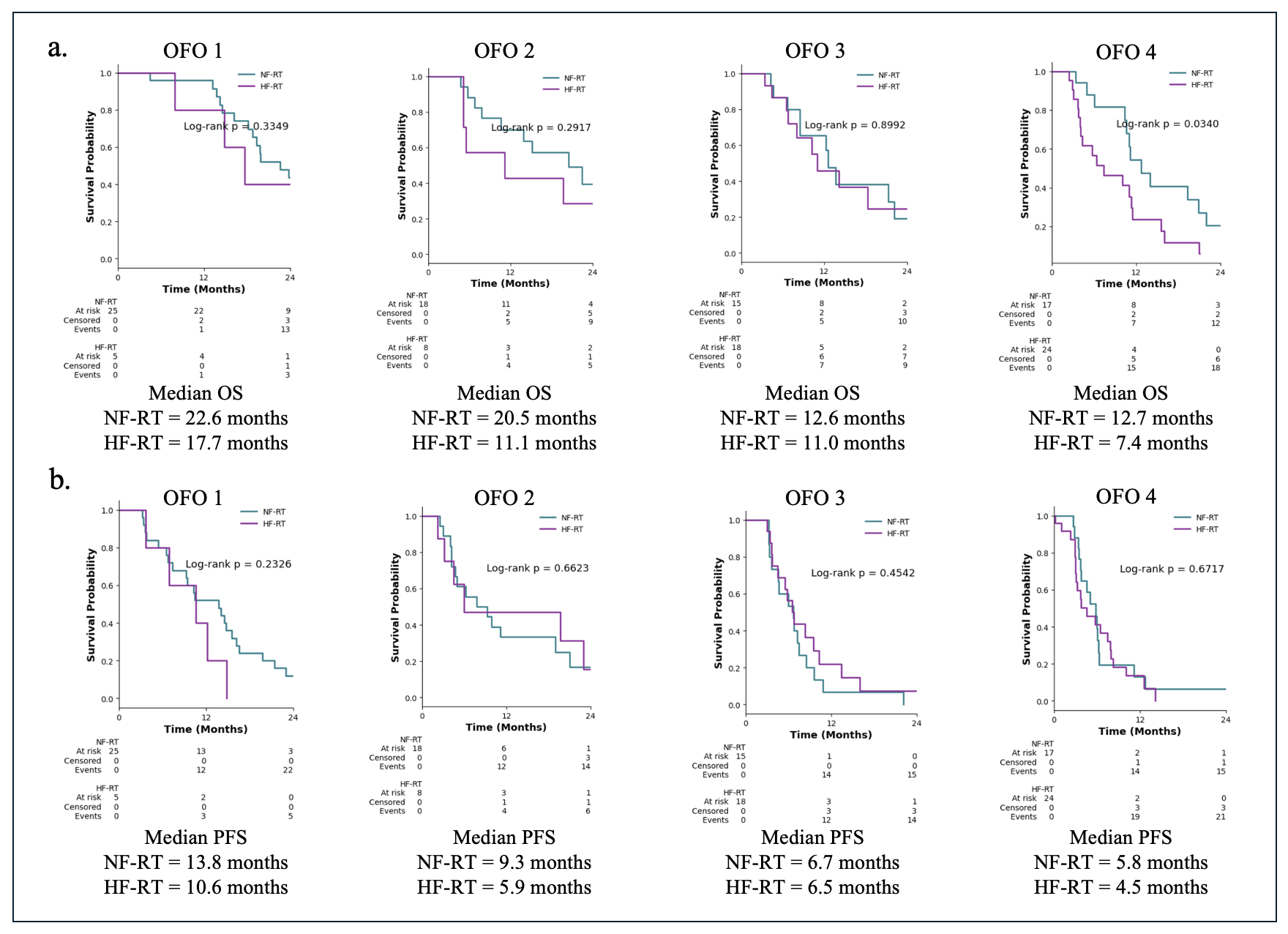


**Figure 3.** Kaplan-Meier survival plot of IDH-wildtype GBM. (a) OS by OFO group 1-4, demonstrating a stepwise decline in OS. (b) PFS by OFO group showing shorter PFS in higher OFO categories. P-values, log-rank test.
